# The Effect of Infrared Drying on Color, Projected Area, Drying Time, and Total Phenolic Content of Rose (*Rose electron*) Petals

**DOI:** 10.3390/plants9020236

**Published:** 2020-02-12

**Authors:** Kemal Çağatay Selvi, Abraham Kabutey, Gürkan Alp Kağan Gürdil, David Herak, Şebnem Kurhan, Pavel Klouček

**Affiliations:** 1 Department of Mechanical Engineering, Czech University of Life Sciences Prague, Kamycka 129, 16521 Prague, Czech Republic; kabutey@tf.czu.cz (A.K.); ggurdil@omu.edu.tr (G.A.K.G.); herak@tf.czu.cz (D.H.); 2 Department of Quality of Agricultural Products, Czech University of Life Sciences, Faculty of Agrobiology, Food and Natural Resources, Kamýcka 129, 16521 Prague, Czech Republic; kurhan@af.czu.cz (Ş.K.); kloucek@af.czu.cz (P.K.)

**Keywords:** infrared drying, rose petals, drying process, total phenolic content

## Abstract

The effects of different drying temperatures (50, 60, 70 °C) on the quality of rose (*Rose electron*) petals were evaluated in this study. Drying time decreased from 1680 s to 600 s with increased infrared temperature. The temperature and time were increased from 50 °C to 70 °C and 30 min to 60 min, respectively, and a decrease in the fruit color quality was observed. The projected area (PA) of rose petals was affected significantly from temperature. After the drying process, the largest PA was observed as 33.35 cm^2^ (50 °C, 30 min), while the smallest achieved at 70 °C, 60 min (27.96 cm^2^). Depending on the temperature values (50, 60, 70 °C), the average projection area of dry samples of the rose petals decreased 2.17 times compared to the projection area of fresh samples. The dried samples demonstrated an increase in the total phenolic (TP) content compared to the fresh samples. The maximum TP (44.49 mg GAE/g) was achieved at 45 min and 70 °C rose petals sample. The results concluded that infrared drying for 45 min at 70 °C could be recommended for drying rose (rosa electron) petals.

## 1. Introduction

The genus Rosa has approximately 100 species that are widely spread out in Europe, Asia, the Middle East, and North America [1], and its flowers in different colors are fragrant and also elegant and beautiful in shape and size. [2]. Today, besides being economically important genera of ornamental horticulture [3], rose is rising as ornamental plants widely grown in the world. They are highly popular as garden ornamental plants and cut flowers [4]. Moreover, medicinal properties, viz. anti-HIV, antibacterial, antiseptic, antioxidant, antiviral, aphrodisiac, antitussive, hypnotic, antidiabetic, relaxant effect on tracheal chains, and a tonic for the heart, liver, stomach [5], and uterus, have been recently reported for this plant, further increasing its demand [6]. Besides, it has an important role in the cosmetics industry, as well [7]. Especially, in the food industry, organic cultured roses are known as edible flowers, and phenolic compounds extracted from this plant have been used to make tea and functional beverages that exert beneficial effects on human health [8]. Considering that medicinal aromatic plants, such as roses, which can be used as a tea, are subjected to some thermal treatments during these processes, it is important to identify optimum conditions for extracting phenolic compounds from roses, due to the use of natural and functional ingredients derived from its flowers. Also, essential oils extracted from rose flowers are important ingredients for perfumes industry [9,10]. In addition to all useful information about this plant, fresh rose flowers are very sensitive and cannot retain their beauty and fresh look for a long time in spite of using the best chemicals for enhancing vase life [11]. A considerable amount of rose flowers waits for a long time until distillation due to the short blooming period and an excessive amount of flowers. There are not only losses of essential oil yield but also losses of quality, as well, depending on the waiting time of petals [12]. Color, which is one of the quality parameters for customers, is a perceptual phenomenon that depends on the observer and the conditions. Kramer [13] stated that the appearance of the product usually determines whether a product is accepted or rejected. Thus, the color of the food surface is the first quality criteria evaluated by consumers and is critical to product acceptance [14,15,16,17]. 

One of the oldest methods known for food preservation is drying. This process can cause changes in some physical properties, such as color, texture, and size. Chemical changes, such as losses of flavor and nutrients, can occur during convective drying, which is commonly used to process fruits and vegetables [18]. Although the two most common methods widely used for drying are sun drying and hot-air drying, they have some disadvantages like the inability to handle large quantities to achieve consistent quality standards, contamination problems, and low energy efficiency, which is not a desirable situation for the food industry [19,20]. While sun and hot-air drying have such disadvantages, infrared heating has advantages over conventional drying under similar drying conditions. Infrared (IR) heating is a potential energy-saving up to 50% [21] and high-efficiency technology, whose application in the food processing field is still progressing [22]. Studies comparing infrared drying with techniques based on air convection showed that the infrared radiation (IR) method is quicker than convection methods [23,24,25]. Various heating studies have been carried out to evaluate the effects of its application for rose drying with solar drying [26], sun drying [11], hot air drying [27], and microwave vacuum drying [28]. Hence, when we look at the rose plant, in particular, it is observed that the studies discussing the effects of thin layer infrared drying method, especially, are very limited or even negligible.

The aims of this study were (1) to observe the effects of different drying temperatures, (2) to determine the color and projected area changing of rose petals and, (3) to find the temperature effects on total phenolic content of the rose petals.

## 2. Materials and Methods

### 2.1. Materials

Petals of rose (*Rose electron*), which is a particularly luminous hybrid tea rose, with very large across (12 cm), full (32-40 petals), high-centered, bright cherry pink flowers were used in this study. Petals were gathered from a botanical garden of the Technical Faculty of CULS University in Prague (Czech Republic, 50°05′16.94″ N–14°25′14.74″ E) and cleaned by removing undesired leaves, sepals, and waste materials. The excess water was removed with the help of a blotter. Only rose petals with healthy structure and appearance were carefully selected and put into the dryer as a thin layer (2.5 g), and the damaged petals were separated manually before putting them into the dryer. Fresh petals were stored in the refrigerator at 4 °C until the drying experiment. 

### 2.2. Drying Experiment

**Figure 1:**
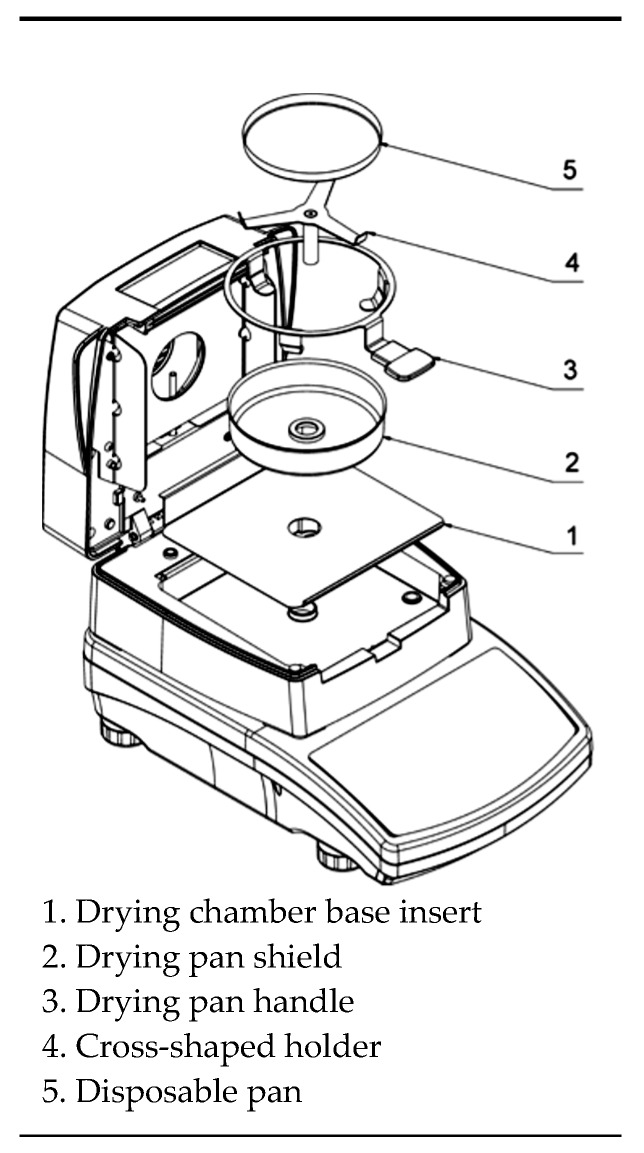
MA.R infrared basic moisture analyzer.

MA.R infrared dryer and moisture analyzer (Radwag balances and scales, Warsaw, POLAND) was used as drying equipment (Figure 1). The drying temperatures were 50, 60, 70 °C in each experiment. The amount of evaporated water was determined during drying at about 2-min intervals at each drying temperature. Tests were replicated three times, and the average weight loss was reported.

### 2.3. Color Measurements

Rose petal color (fresh and dried samples) was measured using a Voltcraft Plus RGB-2000 (Voltcraft, Lindenweg 15, D-92242 Hirschau/GERMANY) color analyzer and described in terms of “L” (Lightness), “a” (redness), and “b” (blueness) values. The instrument was calibrated against a white standard. The average values of color parameters and standard deviations were calculated (L*, a*, b*, C*, and H). The color value of L* showed the brightness, and it ranged from 0 to 100. The values of color coordinates a* and b* did not have a specific measurement range. The sample color meant red if a* value was positive, and it reflected green color when a* value was negative. Besides, if b* value was positive, the color was yellow; if it was negative, the color was described as blue. Metric color chrome C* and hue H* values were calculated using the measured L*a*b* values [29].

C = (a^2^ + b^2^)^1/2^ (1)

h^o^ = tan^−1^ (b/a) (2)

According to Adak, N., et al. [18], total color difference (ΔE) was determined as follows:

∆E = [(L − L_o_)^2^ + (a − a_o_ )^2^ + (b − b_o_ )^2^]^1/2^ (3)

where L_o_, a_o_, and b_o_ indicate the brightness, redness, and yellowness of dried samples, respectively.

### 2.4. Image Analysis to Projected Area

**Figure 2:**
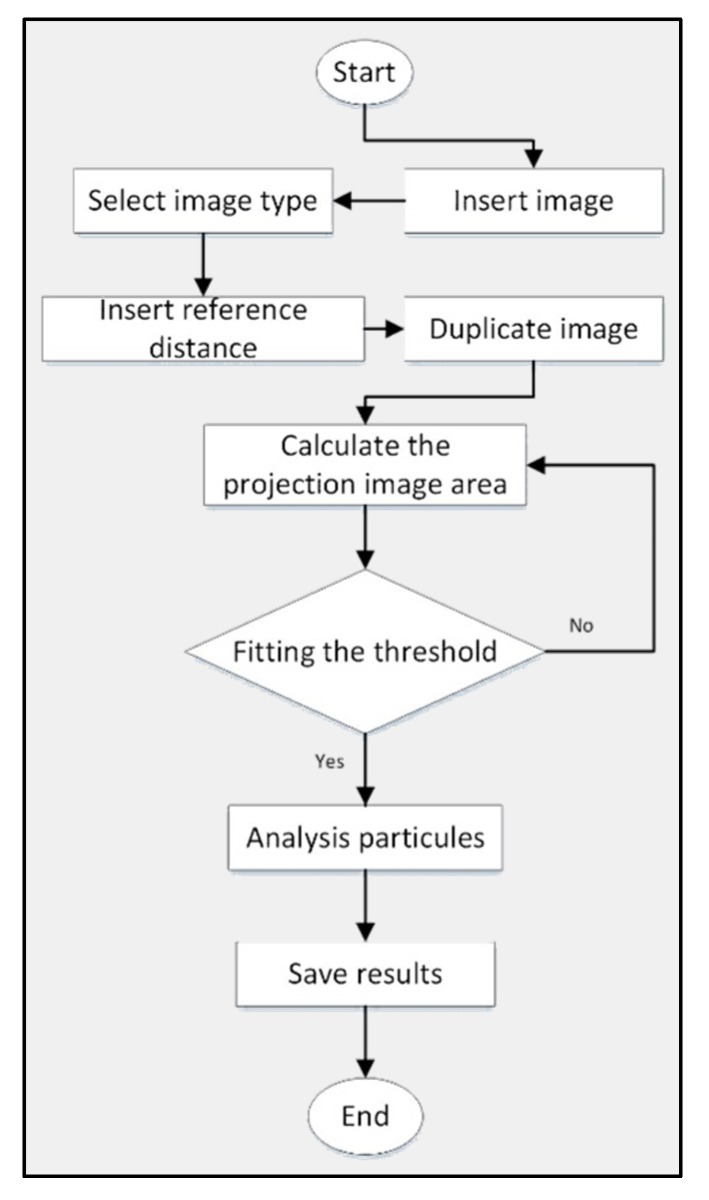
Flowchart of the usage of ImageJ to measure the projected area.

The projected area of the thin layer rose petals was measured using the pixel method in the ImageJ program. ImageJ is Java-based, readily available, open-source, independent platform, and public domain software developed at the National Institutes of Health (NIH), Bethesda, Maryland USA [30]. This method was held at two-dimensional axes (x and y) with reference to the pixel colors. Before and after the drying process, this technique could be used due to being available and being free of charge to be accepted for the indexed journal. The software could measure many parameters of the image analysis, including the projected and surface area of leafy plants like roses. The program uses a threshold-based pixel count measurement to calculate the petals area. Figure 2 illustrates the flowchart of the usage of ImageJ to measure the projected area [31,32]. Nevertheless, the images were taken with the Huawei P20 16 MP camera before and after the drying process in the experiment.

### 2.5. Preparation of Extracts

In order to determine the total phenolic content of the rose samples, dried rose leaves were homogenized. Each dry sample (0.300 g) was weighed into 50 mL screw-capped centrifuge tubes and dissolved in 10 mL of methanol/water (20/80, *v*/*v*) solvent, according to Rusalep et al. [33] with minor modifications. Samples were extracted using an orbital shaker and agitated at 200 rpm for 24 h. To obtain uniform results with dry samples, dry matter content of fresh samples was also determined using a moisture analyzer (KERN, Model DBS 60-3). The obtained extract was used for further analysis. 

### 2.6. Determination of Total Phenolic Content (TPC)

For the determination of TPC by Folin–Cioceltau reagent, the method described by Singleton et al. [34] was used. Briefly, in 96-well plate, a triplicate sample of each 20 µL extract was transferred to the wells and diluted in 180 µL of 20% methanol/water solvent mixture. Using diluted extract, sub-dilutions (x20 and x40, respectively) were prepared in the following wells at a final volume of 100 µL. Subsequently, 25 µL Folin–Cioceltau’s phenol reagents were added, followed by the addition of 75 µL of 12% (*w*/*v*) sodium carbonate to start the reaction. After the appending of the reagents, plates were stored in the dark for 30 min. Absorbance was measured by spectrophotometer (Thermofisher Scientific, Pittsburgh, PA, USA) at 760 nm, and TPC was estimated from an external standard curve of gallic acid. Gallic acid standard calibration range was used between 1.5–100 ppm (R^2^ = 0.9954). TPC of the samples was expressed as gallic acid equivalents (g) per 100 g dry weight (DW) (g of GAE/ 100 g DW). All samples were analyzed in three replicates.

### 2.7. Statistical Analysis

In this study, descriptive statistical methods, such as average and standard error of the mean, were used. Kolmogorov Smirnov test showed that the data were normally distributed (*p* = 0.742), and the Levene test showed that variances were homogeneous (*p* = 0.074); in this case, one-way ANOVA was used to analyze the data [35,36]. The power of the test was found 1.000, and it showed that the sample size was adequate. To compare the means, Duncan multiple comparison test was used. 

## 3. Results and Discussion

### 3.1. Moisture Contents

**Figure 3:**
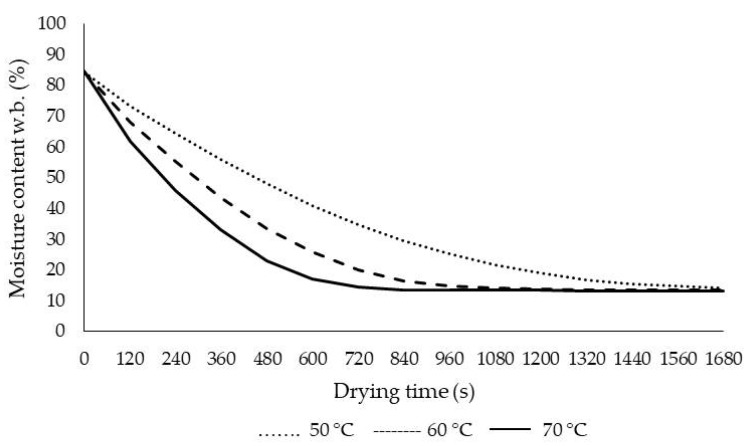
Moisture content changes at different air temperatures.

The drying process (initial moisture content, 85.45% w.b.) was incessant until the final moisture content was ca. 13% (w.b.). Figure 3 shows the changes in moisture content as a function of drying temperature with IR. It was calculated that the drying period reached the above-mentioned safe moisture level at 600, 1080, and 1680 s at 70, 60, and 50 °C, respectively. Drying time reduced dramatically with the increase in infrared temperature. As the temperature increased from 50 °C to 70 °C, the drying time was reduced by about 280%. Balladin and Headley and also Ertekin and Heybeli [24,26] reported that the drying time for rose petals was 16 h for solar drying, and drying time was between 1080 s and 2280 s at drying temperatures of 80–60 °C for mint, respectively. When the effects of temperature on moisture were examined in detail, our results were in line with those for banana slices using infrared and hot air dryer [37], drying tomato under convective infrared drying [38], pulsed vacuum drying, infrared-assisted hot air-drying and hot air-drying for red pepper [39], and nettle leaves under microwave and infrared drying [40,41].

### 3.2. Color

**Figure 4:**
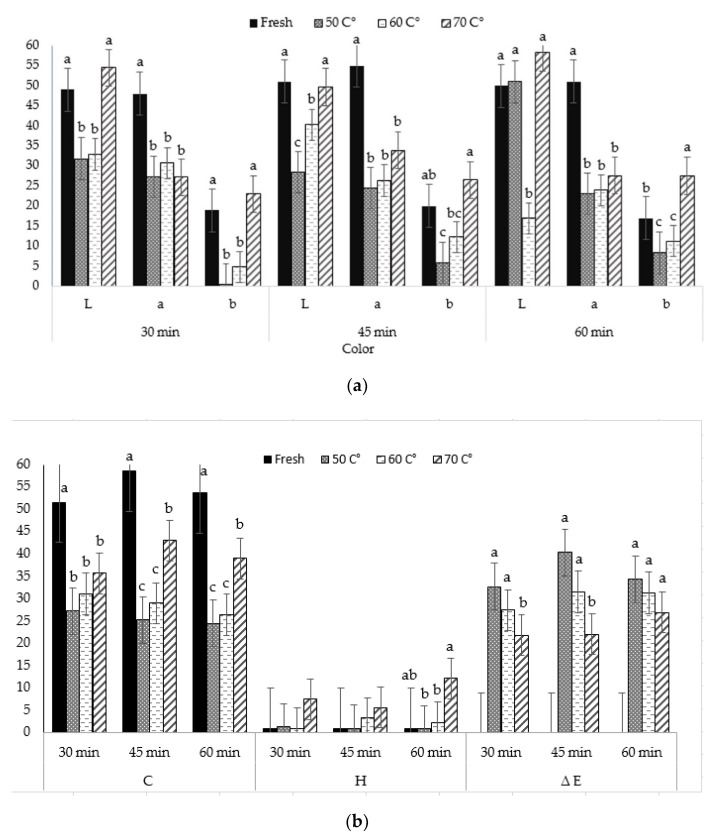
L, a, b (**a**) and C, h, ∆E (**b**) changes at different air temperatures and time. a,b; Different letters of upper index within the column indicate significant differences at *p* < 0.05 level.

The L*, a, b values and, also, C* and h° color properties are presently one of the most popular color indicators for measuring object color and are widely used, virtually, in all fields. The data related to the lightness (L), redness (a), yellowness (b), and also chroma value (C), hue angle (h°), and color difference (ΔE) of fresh and dried petals of *Rose electron* flowers at 50 °C, 60 °C, and 70 °C is presented in Figure 4a,b. In addition, in order to see the effect of different drying times on color with temperature changes, the results were evaluated for three different time periods (30, 45, 60 min). The results showed that significant changes in color values occurred due to temperature change. L, a, b values were higher in fresh petals, in general; but, for b values at 70 °C. With the increase in temperature, the less color was observed. The high temperature yielded in darker, less red, and more brown rose petals. L values ranged from 51.00 to 28.44, a from 55.00 to 23.12, and b from 27.56 to 0.48. So, all drying temperatures decreased brightness (L*), which indicated that fresh petals had a bright color as compared with dried samples. The highest color intensity (C) was 58.52 in fresh fruits. The lowest total color change (ΔE) value was 22.09 at 70 °C. The highest h° value was obtained at 70 degrees as the color difference (ΔE), shown in Table 1. 

Adak, Heybeli, and Ertekin [18] obtained the highest L values at fresh samples under infrared, and, in addition, with the increase in temperature, a and b values decreased first, and when the temperature reached 70 degrees, the values for a and b reached the highest for the strawberry (similar color red and pink). Chen, Gast, and Smithey [14], also found that freeze-drying processes reduced L, a, and b values for red roses and carnations. Bintory, Seetharamu, Munikrishnappa, Ramegowda, and Basavaraj [11] also evaluated air drying and hot air-oven drying in Dutch rose flowers and determined that these drying processes reduced L, C, and h values. Similarly, Siriamornpun, Kaisoon, and Meeso [42] reported smaller L and b values with far infrared-hot air (HA) drying and ∆E in dried marigold petals compared to freeze-drying and HA. In the current study, while the lightness was decreased, the yellowness of the dried rose petals was significantly increased, possibly because of chlorophyll degradation. In another infrared drying study, while the highest C value was observed in fresh strawberry fruit, the lowest h° value was observed at 60 °C [43]. Similarly, in this study, the highest C value was obtained in fresh samples, while the lowest hue angle, defined as color differences, was observed at 70 degrees. On the other hand, the total color difference, ∆E, which is a calculation with L, a, and b values, is a colorimetric parameter widely used to qualify the disparity of color in plants during drying processing. The results showed in our work recommended that the changes in ∆E at 70 °C were smaller as compared to 50 °C and 60 °C. Comparatively and especially, long drying periods were the major factors in color change, resulting in the darkened color [44].

### 3.3. Projected Area (PA)

**Figure 5:**
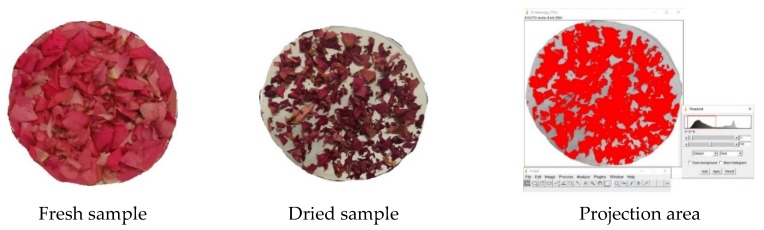
Illustration of projection area variation of rose petals with Image J.

**Figure 6:**
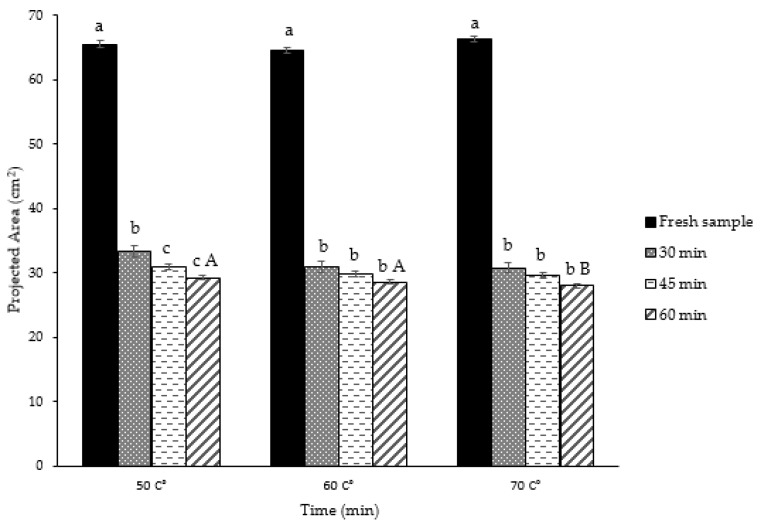
Change and determination of projection areas of fresh thin layer rose petals. a,b,c; Different letters of the upper index within the column indicate significant differences at *p* < 0.05 level. A,B; Different letters of the upper index within the column indicate significant differences at *p* < 0.05 level.

The projected areas of the thin layer rose petals during the IR process by using different temperatures were investigated, as shown in Figure 5. Decreasing of the projected area occurred during the drying of the petals in all temperature treatments. It was shown to be affected by drying air temperature and time for the most part of the drying process (Figure 6). Statistical results (*p* < 0.05) also showed a significant difference between the PA change of petals dried using IR according to temperature. 

Depending on the temperature values (50, 60, 70 °C), the average projection area of dry samples of rose petals decreased 2.17 times compared to the projection area of fresh samples. The largest PA was observed as 33.35 cm^2^ (50 °C, 30 min), while the smallest achieved at 70 °C, 60 min (27.96 cm^2^). At the end of the drying process, based on 50, 60, and 70 °C, the losses in the projection areas of the thin layer rose petals were 50.93%, 47.12%, and 46.97% for 30 min, respectively. The same values were obtained—42.70%, 43.60%, 44.65%—at the end of 60 min. This decrease in the rate of change showed that the effect of temperature during the first 30 min had been realized to a great extent. The very fine structure of the petals and the rapid removal of moisture as a result of this might have caused the shrinkage to occur at the beginning of the drying process. Briefly, this could be due to the combined effect of temperature and drying time on shrinkage. Similar statements that drying reduces the projection area of leaves [45] and similar effects of drying air temperature and time on the projected area for different agricultural crops have been reported in the literature [41]. 

### 3.4. Total Phenolic Content (TPC)

The total phenolic content of the rose petals extracts dried at different temperature-time combinations was determined by the Folin–Cioceltau colorimetric method. Table 2 presents the TPC of the rose petals dried using different temperatures. The drying temperatures affected the TPC. 

The fresh sample was initially measured at 1.21g GAE/100 g for the TPC. The TPC in the dried samples (for 50 °C, 60 °C, 70 °C) was significantly (*p* < 0.05) higher than that in the fresh. The content of total phenolic varied from 1.29 g GAE/100 g (Fresh sample) to 5.11 gGAE/100 g of d.w. Drying at 50 °C for 30 min yielded the lowest concentration of total phenolic in comparison to a higher temperature and/or longer time drying conditions. The increase of the TPC along with the extended drying duration at 50 °C might relate to the increased inactivation of enzymes, which are natural constituents of plant cells and responsible for the degradation of the phenolic compounds. As reported by Cheng at al. [46], polyphenol oxidase enzymes are resistant to mild heat treatments, especially when the drying temperature is below 55 °C. The sample from 45 min and 70 °C resulted in the highest TPC in the rose petals (5.11 g GAE/100 g). The same trend was observed by Chang, Lin, Chang, and Liu [47] for tomatoes, Honarvar et al. [48] for Rosa moschata, and Da Silva et al. [49] for pineapple when they evaluated the content of phenolic compounds after drying; they also obtained higher values of TPC in the dried samples than in the fresh samples. Similar findings were also observed by Saifullah et al. [50] during drying lemon myrtle with hot air at 50, 70, and 90 °C for 315, 105, and 75 min, respectively. They obtained the highest total phenolic content at 90 °C for 75 min and suggested that drying at higher temperatures requires shorter times in order to avoid phenolic and other phytochemical content degradation. The TPC of the 45 min and 70 °C rose petals sample was 3.95 times higher than that of the fresh sample and 1.54 times higher than that of the 50 °C sample (2.61 g GAE/100 g) and 1.33 times higher than 60 °C (2.92 g GAE/100 g) for 45 min. The results showed statistically significant changes (*p* > 0.05) between the TPC values of the time changing. Heating at 50 °C, 60 °C, and 70 °C for different times increased the total phenolic content for rose petals. This result showed that the IR drying of rose petals promoted the increase in the amounts of total phenols extracted from the petals. This observation could be referred to the fact that IR drying ruins the petals’ cell walls and thus facilitate the disentanglement of the phenol compounds. On the other hand, the higher temperature might have affected the retention of phenolic compounds from the cells due to disruption of cell integrity, and consequently release of more phenolic compounds, as well as resulted in efficient enzyme inactivation. Another prospect related the low level of phenolic compounds in the fresh sample to the existence of an active enzyme in the sample that presumably caused these compounds to be destroyed. In the dried samples, by virtue of the low water activity, devastating enzymes were inactivated, and high levels of phenolic compounds remained in the extract [51] (Hassain et al. 2010). Similarly, Prathapan et al. [52] studied the temperature effect (60–100 °C) on TPC, and, in their study, TPC values increased gradually when samples were heated from 60 to 80 °C. Moreover, polyphenol oxidase PPO was completely inactivated at 80 °C. In another congener study, the increase of total phenols in dried olive leaves was also sighted by Boudhrioua et al. [53] for infrared dried olive leaves. The researchers studied the effect of different infrared drying temperatures (40 °C 50 °C, 60 °C, and 70 °C) on total phenol contents and color of four varieties of olive leaves. The authors declared that the drying processes increased the TPC of olive leaves. Similar results were also reported by Ngamsuk et al. [54] and Chang et al. [47] and for grape pomace by Cascaes-Teles et al. [55]. Drying treatments have various effects on phenolic compounds, as stated by these observations, but it would be good to emphasize another issue here that the total phenolic content of samples is just indicative. Chemical changes during the drying process should be done by HPLC instead of Folin–Ciocalteu method for further and detailed investigation. On the other hand, the functional properties of food drying that are influenced by the intricate chemical interactions are still under research. 

## 4. Conclusions

The aim of the present research was to examine the effect of different heating temperatures in infrared drying on color, projected area, drying time, and total phenolic content of rose (*Rose electron*) petals. For this purpose, thin-layer infrared drying experiments for rose petals were conducted at specific drying temperatures of 50, 60, 70 °C. The results showed that significant changes in color values occurred due to temperature change. The highest b values were obtained at 70 °C at 60 min of drying, while the lowest ∆E was achieved at this temperature. 

When compared to fresh samples, the projection area reduced by approximately half at all treatments. These experiments confirmed that the drying time decreased when compared with convective, freeze, heat pump, solar, and natural sun drying. 

The investigation of temperature effect on TPC showed that the TPC increased with increasing temperatures. The evidence from this study suggested that the IR drying process for 45 min at 70 ℃ could be recommended in terms of TPC for this type of rose. Also, considering the further investigation in terms of total phenolic content, more detailed results could be obtained with HPLC analysis instead of the Folin–Ciocalteu method.

## Figures and Tables

**Table 1:** Color parameter changing at different air temperatures and time (Mean ± Standard Error).

	Fresh Leaves					
**Time (min)**	**L**	a*	b*	**C**	**h**	**∆E**
30	49.00 ± 3.99 ^a^	48.00 ± 3.88 ^a^	19.00 ± 1.21 ^a^	51.62 ± 4.33 ^a^	1.00 ± 0.09	-
45	51.00 ± 4.21 ^a^	55.00 ± 4.01 ^a^	20.00 ± 1.15 ^ab^	58.52 ± 4.12 ^a^	1.00 ± 0.09	-
60	50.00 ± 3.80 ^a^	51.00 ± 3.98 ^a^	17.00 ± 1.23 ^b^	53.76 ± 3.99 ^a^	1.00 ± 0.08 ^a b^	-
	**50 °C**					
**Time (min)**	**L**	a*	b*	**C**	**h**	**∆E**
30	49.00 ± 2.99 ^b^	27.25 ± 2.10 ^b^	0.48 ± 0.03 ^b^	27.25 ± 1.78 ^b^	1.33 ± 0.10	32.69 ± 1.18 ^a^
45	49.69 ± 3.29 ^c^	24.58 ± 1.85 ^b^	5.89 ± 0.04 ^c^	25.28 ± 1.99 ^c^	1.000 ± 0.086	40.42 ± 3.56 ^a^
60	49.00 ± 4.12 ^a^	23.12 ± 1.87 ^b^	8.29 ± 0.06 ^c^	24.56 ± 2.15 ^c^	0.8600 ± 0.0072 ^b^	31.58 ± 2.99 ^a^
	**60 °C**					
**Time (min)**	**L**	a*	b*	**C**	**h**	**∆E**
30	32.90 ± 2.28 ^b^	30.77 ± 2.98 ^b^	4.8 ± 0.04 ^b^	31.14 ± 2.58 ^b^	1.000 ± 0.069	27.53 ± 245 ^a^
45	40.25 ± 3.13 ^b^	26.35 ± 1.88 ^b^	12.32 ± 0.9 ^bc^	29.09 ± 2.49 ^c^	3.33 ± 0.21	31.55 ± 2.88 ^a^
60	45.00 ± 4.08 ^b^	23.97 ± 1.17 ^b^	11.27 ± 0.85 ^c^	26.49 ± 2.14 ^c^	2.33 ± 0.18 ^b^	28.08 ± 2.46 ^a^
	**70 °C**					
**Time (min)**	**L**	a*	b*	**C**	**h**	**∆E**
30	31.83 ± 1.19 ^a^	27.28 ± 1.75 ^b^	23.07 ± 2.21 ^a^	35.73 ± 2.69 ^b^	7.60 ± 0.55	27.22 ± 1.98 ^b^
45	28.44 ± 1.10 ^a^	33.93 ± 1.98 ^b^	26.51 ± 1.18 ^a^	43.06 ± 3.77 ^b^	5.67 ± 0.38	22.09 ± 1.82 ^b^
60	38.00 ± 2.18 ^a^	27.61 ± 2.12 ^b^	27.56 ± 1.65 ^a^	39.01 ± 3.25 ^b^	12.20 ± 0.95 ^a^	25.68 ± 2.41 ^a^

a,b,c; Different letters of the upper index within the column indicate significant differences at *p* < 0.05 level. L; lightness, a; redness, b; yellowness, C; chroma value, h°; hue angle.

**Table 2:** TPC (total phenolic content) of rose petals under different times and temperatures.

No.	Sample	Amount of TPC (gGAE/100 g)	Standard Error
**1**	Fresh sample	1.29 ^g^*	0.05
**2**	30 min-50 °C	1.89 ^f^	0.14
**3**	30 min-60 °C	4.19 ^bc^	0.32
**4**	30 min-70 °C	4.45 ^bc^	0.17
**6**	45 min-50 °C	3.33 ^e^	0.22
**7**	45 min-60 °C	3.84 ^d^	0.10
**8**	45 min-70 °C	5.11 ^a^	0.13
**10**	60 min-50 °C	3.41 ^e^	0.28
**11**	60 min-60 °C	4.14 ^bc^	0.40
**12**	60 min-70 °C	4.14 ^c^	0.14

* Values in the same column that are followed by different letters are significantly different (*p* < 0.05) using Duncan comparison test.

## Abbreviations

C	degree (°)
PA	projected area (mm^2^)
TPC	total phenolic content (mg GAE/g)
GAE	gallic acid equivalent
L	lightness
a	redness
b	blueness
C*	chrome
H*	hue angle
∆E	color difference
